# Estimating the effect of physical activity on cognitive function within the UK Biobank cohort

**DOI:** 10.1093/ije/dyad009

**Published:** 2023-02-07

**Authors:** Thomas Campbell, Breda Cullen

**Affiliations:** School of Health and Wellbeing, University of Glasgow, Glasgow, UK; NHS Lanarkshire Neuropsychology Service, Monklands Hospital, Airdrie, UK; School of Health and Wellbeing, University of Glasgow, Glasgow, UK

**Keywords:** Physical activity, cognitive function, directed acyclic graph, healthy ageing, UK Biobank

## Abstract

**Background:**

Physical activity (PA) has been associated with benefits for cognitive function (CF), but previous estimates of the strength of this relationship may have been biased due to limitations in statistical modelling practices that are common among observational studies. We aimed to address this by using a rigorously constructed conceptual causal model to guide an empirical analysis estimating the effect of PA on CF in the UK Biobank cohort of middle-aged and older adults.

**Methods:**

This study analysed a subsample of 334 227 adults from the UK Biobank prospective cohort study. PA was measured subjectively by self-report and by device using accelerometry, and CF was measured using objective cognitive tests. Composite CF measures were derived to represent general and domain-specific performance. Effect coefficients were estimated using regression models, adjusting for a wide range of confounders specified by the assumed causal model, including genetic risk factors, and relevant health, sociodemographic and behavioural variables from across the lifespan.

**Results:**

Results indicated very small effect sizes (standardized mean difference estimates all <0.01) of inconsistent direction, for both cross-sectional and longitudinal analyses.

**Conclusions:**

The expected protective effect of PA on CF was not observed. This may reflect selection bias within UK Biobank, or the relatively young age of the sample at follow-up.

Key Messages

- We present an empirical analysis of the UK Biobank cohort investigating the association between physical activity and cognitive function.
- A rigorously constructed directed acyclic graph was used to guide statistical modelling decisions and address common methodological limitations in observational studies.
- In contrast to the majority of previous literature, the association between physical activity and cognitive function was virtually zero in both unadjusted and adjusted models.
- We discuss potential reasons for this finding, including the role of selection bias within the UK Biobank sample, and reverse causality in previous studies of physical activity and cognitive function.

## Introduction

The term cognitive function (CF) describes the set of mental abilities that enable the acquisition and use of knowledge and skills throughout life. It is recognized that CF is multidimensional and consists of abilities within subdomains such as memory, speed of processing, verbal ability and reasoning, and that abilities within these domains tend to be positively correlated.^1^ Brain imaging studies show associations between various characteristics of the brain, such as grey and white matter volumes, and CF.^2^

Physical activity (PA) is defined as ‘any bodily movement produced by skeletal muscles that requires energy expenditure’.^3^ PA thus includes everyday activities as well as purposeful exercise. Researchers typically conceptualize PA along a continuum, and tools such as the International Physical Activity Questionnaire (IPAQ) categorize activities according to their intensity as ‘light’, ‘moderate’ and ‘vigorous’.^4^ The World Health Organization (WHO) recommends that adults should undertake 150–300 min of moderate intensity, or 75–150 min of vigorous physical activity, per week.^5^

Recent evidence appears to converge at the view that PA is associated with benefits for CF, whether CF is conceptualized globally or at subdomain level.^6–9^ However, due to the observational nature of such studies, researchers have been cautious about making causal claims from their findings. Furthermore, the statistical adjustment strategies used in such studies often neglect to consider the assumptions behind, and implications of, the selection of variables included for adjustment, and thus may introduce more bias into effect estimates.^10^

To address these potential shortcomings in the existing literature, we conducted a systematic review^11^ using a protocol^12^ for synthesizing observational evidence to produce a directed acyclic graph (DAG). This involved integrating relevant covariates identified by the review into a model by mapping the assumed structure of inter-relationships between these variables, using a set of causal criteria to guide decisions. This enabled us to identify confounders and mediators of the PA-CF relationship and select appropriate adjustment strategies. There was a large number of mediators and confounders indicated by this process. Intermediate mechanisms by which PA may affect CF include by facilitating neurogenesis, synaptogenesis and angiogenesis^13^ and modifying grey matter volume^14^ and white matter integrity.^15^ Identified confounders broadly fell into the following categories: pre-birth factors (e.g. genetic risk); early life factors (e.g. childhood PA, education, traumatic events); adult sociodemographic factors (e.g. socioeconomic status, exposure to pollution); adult health behaviours (e.g. diet, alcohol consumption and smoking status); adult health outcomes (e.g. cardiovascular disease, neurological disease, mental health disorders); and medication (e.g. psychotropic or antihypertensive medication).

Previous observational studies of the PA-CF relationship may be biased because none has followed a comprehensive method to select covariates. Our study addressed this by using the DAG produced by our review^11^ to inform the specification of models to estimate the total effect of PA on CF (i.e. including any effect transmitted indirectly via intermediate variables). This study matched variables from the DAG with data available within the UK Biobank dataset, in order to address the following research aims:

- To estimate the magnitude of the relationship between PA and CF in a cross-sectional analysis of baseline UK Biobank data;
- To estimate the magnitude of the relationship between PA at baseline and CF at follow-up in a longitudinal analysis.

## Methods

This study is reported according to the Strengthening the Reporting of Observational Studies in Epidemiology (STROBE) statement.^16^

### Participants

UK Biobank is a very large prospective cohort study of over 500 000 participants, designed to examine the genetic and environmental determinants of health in middle-aged to older adults in the general population.^17^ Our study was conducted under approved application 11332. Participants provided written informed consent. Recruitment was based on proximity to an assessment centre and being within the eligible age range of 40–69.

**Figure 1:**
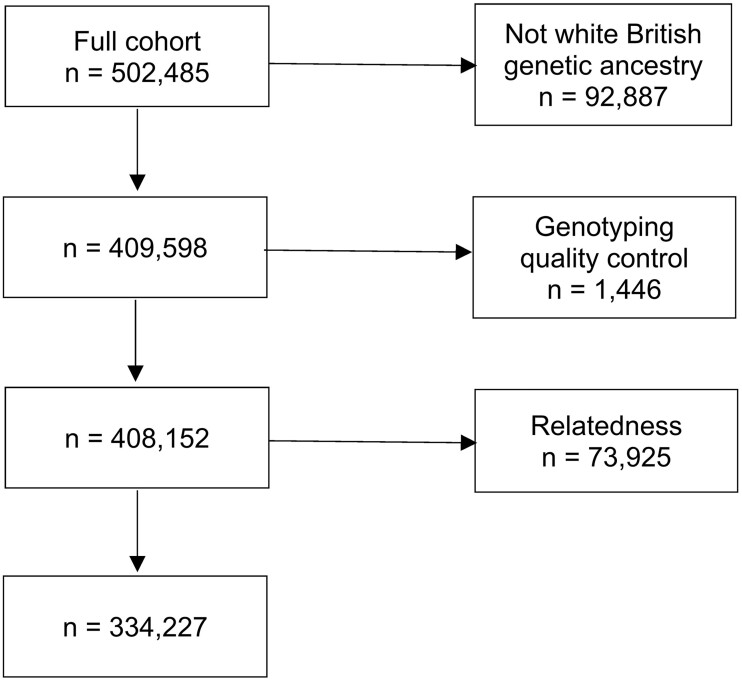
Flowchart showing participants included in the study

During the baseline assessment (2006–10), participants attended assessment centres around the UK where they completed self-report sociodemographic, health and lifestyle questionnaires and an interview with a trained staff member, as well as undergoing physical and biological measurements and a brief computerized cognitive assessment. Subsequently, a subset of the total cohort completed accelerometry (device-measured PA) and neuroimaging visits (including repeat cognitive assessments). Invitations to participate in accelerometry (2013–15)^18^ were sent to a random sample of participants with e-mail addresses (excluding those closest to the main UK Biobank centre, due to concerns about burden on those participants). Invitations to the neuroimaging assessments (2014 onwards) were based on proximity to UK Biobank MRI scanning centres in England. Because our study made use of genetic score data, the analysis sample was restricted to those with White British genetic ancestry (as determined centrally by UK Biobank based on a combination of self-report and genetic data) in order to reduce confounding induced by groups differing systematically both by genetic ancestry and according to phenotypic measures of interest.^19^ This type of restriction has been performed in UK Biobank studies previously.^20^ Similarly, the sample was restricted to unrelated people; this was done by randomly keeping one member of each related set (third degree or closer). Along with further standard exclusions based on genotyping quality control, this left a sample of 334 227 which was used for baseline analysis in our study (see Figure 1).

## Measures

The variables within the conceptual DAG model^11^ were matched to the data available within UK Biobank (see Supplementary Methods, available as Supplementary data at *IJE* online for detail).

### Exposure: baseline physical activity

Self-report data: at the assessment centre visits, participants completed a modified form of the IPAQ short form,^21^ reporting the frequency and duration of walking and moderate and vigorous activity undertaken in a typical week. Data were processed in accordance with IPAQ scoring guidance, such that each category of activity was assigned the following weighting in metabolic equivalent of task (MET) units: walking, 3.3 METs; moderate activities, 4 METs; and vigorous activities, 8 METs. Total amount of moderate-vigorous PA was estimated as the sum of these moderate and vigorous PA expressed in MET-hours per week, and classified as active if they met IPAQ recommendations of at least 10 MET-hours per week of moderate to vigorous PA, as has been done in previous UK Biobank studies.^22^ Total PA was calculated by summing the weighted time spent across all three categories and expressed in MET-hours per week. Therefore, participants received a total PA value if they had data for at least one of the three levels of activity.

#### Accelerometer data

A subsample participated in accelerometer-measured PA data collection for a 1-week period. The Axivity AX3 wrist-worn triaxial device recorded accelerations over 5-s periods (measured in milligravity, mg, units), resulting in 120 960 data points per participant.^18^ Mean daily accelerations per day were derived for use in analysis. The levels of missing data and overall participant compliance in collecting these data are similar to other studies.^18^

### Outcome: cognitive function

Cognitive tests have been administered at several time points to UK Biobank participants. These tests were developed specifically for UK Biobank, to enable computerized administration at scale without staff involvement, and are thus non-standardized. However, the psychometric properties of these measures have since been compared with well-validated reference tests.^23^

#### Baseline cognitive function tests

During the original baseline assessment, almost all participants completed touchscreen tests of visuospatial memory (‘Pairs Matching’) and processing speed (‘Reaction Time’). Additional tests of prospective memory (‘Prospective Memory’), attention/working memory (‘Numeric Memory’) and verbal and numerical reasoning (‘Reasoning’) were added to the battery part way through the recruitment period, one of which (Numeric Memory) was subsequently removed for reasons of time. This and missing values throughout the dataset, resulted in different sample sizes for analysis. For baseline analyses, sample sizes ranged from 31 854 (Numeric Memory) to 305 294 (Reaction Time) in unadjusted models. Adjusted model sample sizes ranged from 2548 (Prospective Memory) to 29 810 (Reaction Time). For further detail on each test, see Supplementary Methods.

#### Follow-up cognitive function tests

The follow-up data in our study pertain to 10 tests administered at the imaging visit, which included the five administered at baseline as well as the following five: ‘Trail Making Test’ (Part A reflects processing speed, Part B executive function); ‘Digit Symbol Substitution Test’ (processing speed); ‘Tower Rearranging Test’ (executive function); ‘Paired Associate Learning’ (verbal declarative memory); ‘Matrices’ (non-verbal reasoning). For further detail, see Supplementary Methods.. One additional test (‘Picture Vocabulary’) was administered at the imaging visits, but the data from this test have not yet been released by UK Biobank and so this is not described further here. For longitudinal models using self-reported PA, sample sizes ranged from 21 225 (Trails-B Time) to 30 330 (Prospective Memory). After adjusting the models for the specified covariates, sample sizes ranged from 4805 (Paired Associate Learning) to 6840 (Prospective Memory). Longitudinal models using accelerometer-measured PA ranged from 9362 (Trails-B errors) to 14 392 (Prospective Memory) and, after adjusting for specified covariates, from 2742 (Trails-B Time) to 3935 (Reaction Time).

#### Individual and composite cognitive function scores

For our study, the raw scores for all tests except Prospective Memory (as it is a binary variable) were converted into z-scores for ease of interpretation, standardized within 5-year age bands at each assessment time point. Therefore the mean score is approximately zero, and the standard deviation is approximately one. For each z-score, higher scores represent better performance.

Composite measures of global CF at baseline and at the imaging visit were derived by taking the mean of the four baseline visit z-scores and the 10 imaging visit z-scores (trails B-A not included), for participants with at least two non-missing z-score values. As the imaging visit data included multiple tests that measure the same domain of CF, domain-level composite scores were also derived using the mean of z-scores for participants who had at least two non-missing values for tests within that domain: processing speed (Digit Symbol Substitution and Reaction Time); reasoning (Reasoning and Matrices); executive function (Tower Rearranging, Trails-A time and Trails-B time); and memory (Pairs Matching, Numeric Memory and Paired Associate Learning).

### Covariates

The covariates were identified by the graphical causal model reported in the systematic review^11^ and matched to available UK Biobank data. These are described in the Supplementary Methods and listed in full in Table 1.

### Unmatched variables

There were several variables within the conceptual model which could not be matched to data within UK Biobank. These were childhood PA, childhood intelligence quotient (IQ), earlier adulthood PA and cognitive activity. Of these, childhood PA and earlier adulthood PA were specified in all minimum covariate adjustment sets determined by structural analysis of the DAG. Thus, the model estimated in this study represents the nearest approximation of the full conceptual model, as is recommended in recent guidance.^24^

### Statistical analysis

All analyses were performed in Stata version 16. Data were summarized using descriptive statistics and are reported for the whole sample and stratified by PA classification: active, inactive or missing. Normally distributed continuous variables are summarized as means and standard deviations, and skewed variables as medians with interquartile ranges. Ordinal and binary variables are reported as frequencies and percentages within each category. These summary statistics are presented for the baseline characteristics of the total sample and the subsample who attended the imaging visit (Table 1a). Data pertaining to the cognitive outcomes at follow-up are presented in Table 1b. Differences between the PA groups for each measure were not formally tested, as the decision about entering covariates into the regression models was based a priori on the DAG rather than on the existence of statistical differences. The relationship between PA and CF was then estimated using two sets of regression models using the ‘regress, vce(robust)’ command in Stata: the entire analysis code file is available at [https://osf.io/tngqh/].

**Table 1a:** Baseline characteristics of total sample and imaging subsample

Total sample					Imaging sub-sample (at baseline)			
	Total sample	Active (≥10 MET hours mvPA/week)	Inactive (<10 MET hours of mvPA/week)	Missing PA status^a^	Total sample	Active (≥10 MET hours mvPA/week)	Inactive (<10 MET hours of mvPA/week)	Missing PA status^a^
***n* (%) of sample**	334 227 (100.00)	181 587 (54.33)	82 917 (24.81)	69 723 (20.86)	34 058 (100.00)	19 495 (57.24)	9175 (26.94)	5338 (15.82)
**Demographics**					**Demographics**			
**Age (years)**					**Age (years)**			
Mean (SD)	56.86 (7.99)	56.80 (8.14)	56.49 (7.84)	57.46 (7.70)	55.46 (7.50)	55.57 (7.64)	55.30 (7.28)	55.35 (7.31)
**Sex**								
*n* (%) female	179 421 (53.68)	94 029 (51.78)	44 798 (54.03)	40 594 (58.22)	17 317 (50.85)	9598 (49.23)	4682 (51.03)	3037 (56.37)
**Physical activity**					**Physical activity**			
**Acceleration average (milligravity units)**					**Acceleration average (milligravity units)**			
*n* (%) missing	262 062 (78.41)	140 156 (77.18)	63 969 (77.15)	57 937 (83.10)	18 837 (55.31)	10 606 (54.40)	5187 (56.53)	5388 (56.50)
Mean (SD)	27.41 (14.65)	28.75 (16.17)	25.95 (13.84)	25.09 (8.54)	28.08 (9.08)	29.26 (9.60)	26.60 (8.03)	26.08 (7.91)
**mvPA, self-report (MET hours/week)**					**mvPA, self-report (MET hours/week)**			
*n* (%) missing	69 723 (20.86)	0 (0)	0 (0)	69 723 (100.00)	5388 (15.82)	0 (0)	0 (0)	5388 (100.00)
Median	18.67	30.00	4.00	–	18.00	28.00	4.00	–
(Q1, Q3)	(8.00, 40.00)	(18.00, 56.00)	(2.00, 6.67)	–	(7.33, 36.00)	(17.33, 36.00)	(2.00, 6.67)	–
**Total PA, self-report (MET hours/week)**					**Total PA, self-report (MET hours/week)**			
*n* (%) missing	27 310 (8.17)	0 (0)	0 (0)	27 310 (39.17)	1803 (5.29)	0 (0)	0 (0)	1803 (33.46)
Median	28.22	47.55	11.90	8.25	27.55	43.62	11.70	7.70
(Q1, Q3)	(12.89, 58.10)	(29.30, 84.00)	(7.26, 19.00)	(4.13, 16.50)	(13.20, 53.10)	(27.95, 73.7)	(7.26, 18.50)	(3.30, 15.40)
**Cognitive function (baseline tests)**					**Cognitive function (baseline tests in imaging subsample)**			
**Numerical memory (z score)**					**Numerical memory (z score)**			
*n* (%) missing	299 740 (89.68)	161 673 (89.03)	74 727 (90.12)	63 340 (90.85)	30 634 (89.95)	17 460 (89.56)	8283 (90.27)	4892 (90.79)
Mean (SD)	−0.35 (0.94)	−0.34 (0.93)	−0.27 (0.93)	−0.46 (0.94)	−0.18 (0.93)	−0.20 (0.93)	−0.13 (0.93)	−0.20 (0.93)
**Pairs matching (z score)**					**Pairs matching (z score)**			
*n* (%) missing	7550 (2.26)	3385 (1.86)	1472 (1.78)	2693 (3.86)	446 (1.31)	179 (0.92)	70 (0.76)	197 (3.66)
Mean (SD)	0.24 (1.03)	0.23 (1.04)	0.27 (1.03)	0.22 (1.04)	0.32 (1.03)	0.31 (1.03)	0.33 (1.03)	0.35 (1.01)
**Prospective memory**					**Prospective memory**			
*n* (%) missing	223 812 (66.96)	181 587 (65.90)	55 812 (67.31)	48 330 (69.32)	22 689 (66.62)	12 831 (65.82)	9175 (66.79)	3730 (69.23)
*n* (%) correct on first attempt	88 383 (80.05)	49 673 (80.23)	22 505 (83.03)	16 205 (75.75)	8551 (87.68)	5809 (86.73)	2742 (89.75)	1492 (85.21)
**Reaction time (z score)**					**Reaction time (z score)**			
*n* (%) missing	2010 (0.60)	849 (0.47)	427 (0.51)	734 (1.05)	65 (0.19)	25 (0.13)	18 (0.20)	22 (0.41)
Mean (SD)	0.05 (0.95)	0.08 (0.94)	0.06 (0.94)	−0.02 (0.96)	0.16 (0.93)	0.18 (0.92)	0.14 (0.92)	0.11 (0.93)
**Reasoning (z score)**					**Reasoning (Z score)**			
*n* (%) missing	1363 (1.24)	646 (1.05)	226 (0.84)	491 (2.33)	22 689 (66.62)	12 831 (65.82)	9175 (66.79)	3730 (69.23)
Mean (SD)	−0.13 (0.94)	−0.13 (0.93)	0.01 (0.93)	−0.28 (0.94)	0.14 (0.89)	0.10 (0.90)	0.24 (0.87)	0.08 (0.90)
**Global CFb (z score)**					**Global CF** ^b^ **(z score)**			
*n* (%) missing	7540 (2.26)	3324 (1.83)	1462 (1.76)	2754 (3.95)	2370 (6.96)	1407 (7.22)	539 (5.87)	424 (7.87)
Mean (SD)	0.11 (0.70)	0.11 (0.69)	0.14 (0.69)	0.05 (0.71)	−0.06 (0.46)	− 0.06 (0.46)	−0.03 (0.45)	−0.11 (0.46)
**Genetics**					**Genetics**			
**APOE genotype**					**APOE genotype**			
*n* (%) missing	0 (0)	0 (0)	0 (0)	0 (0)	0 (0)	0 (0)	0 (0)	0 (0)
*n* (%) with each number of APOE e4 alleles								
0	237 761 (71.14)	128 621 (70.83)	59 227 (71.43)	49 913 (71.59)	24 527 (72.02)	14 036 (72.00)	6623 (72.19)	3868 (71.79)
1	88 276 (26.44)	48 461 (26.69)	21 703 (26.17)	18 212 (26.12)	8754 (25.70)	4998 (25.64)	2343 (25.54)	1413 (26.22)
2	8090 (2.42)	4505 (2.48)	1987 (2.40)	1598 (2.29)	777 (2.28)	461 (2.36)	209 (2.08)	107 (1.99)
**Polygenic dementia risk score (z score)**					**Polygenic dementia risk score (z score)**			
*n* (%) missing	1000 (0.30)	545 (0.30)	242 (0.29)	213 (0.31)	113 (0.33)	65 (0.33)	31 (0.34)	17 (0.32)
Mean (SD)	0.00 (1.00)	0.00 (1.00)	0.00 (1.00)	0.00 (1.00)	−0.02 (1.00)	−0.02 (1.00)	−0.01 (1.00)	−0.01 (1.00)
**Familial risk**					**Familial risk**			
**Dementia (parent or sibling with diagnosis)**					**Dementia (parent or sibling with diagnosis)**			
*n* (%) missing	48 778 (14.59)	24 599 (13.55)	10 916 (13.16)	13 263 (19.02)	3954 (11.61)	2198 (11.27)	1023 (11.15)	733 (13.60)
*n* (%) with diagnosis	40 168 (12.02)	21 851 (12.03)	10 123 (12.21)	9194 (11.75)	4168 (12.24)	2384 (12.23)	1147 (12.50)	637 (11.82)
**Maternal smoking around birth**					**Maternal smoking around birth**			
*n* (%) missing	46 978 (14.06)	24 009 (13.22)	11 214 (13.52)	11 755 (16.86)	4127 (12.12)	2281 (11.70)	1079 (11.76)	767 (14.24)
*n* (%) answered yes	88 016 (26.33)	47 858 (26.36)	21 284 (25.67)	18 874 (27.07)	9119 (26.77)	5210 (26.72)	2420 (26.38)	1489 (27.64)
**Parkinson’s disease (parent or sibling with diagnosis)**					**Parkinson’s disease (parent or sibling with diagnosis)**			
*n* (%) missing	53 562 (16.03)	26 860 (14.79)	12 045 (14.53)	14 657 (21.02)	4205 (12.35)	2289 (11.74)	1081 (11.78)	835 (15.50)
n (%) with diagnosis	13 606 (4.07)	7272 (4.00)	3451 (4.16)	2833 (4.13)	1362 (4.00)	792 (4.06)	359 (3.91)	211 (3.92)
**Sociodemographic**					**Sociodemographic**			
**Acculturation (years in UK)**					**Acculturation (years in UK)**			
*n* (%) missing	0 (0)	0 (0)	0 (0)	0 (0)	0 (0)	0 (0)	0 (0)	0 (0)
Median (Q1, Q3)	58.00 (50.00, 63.00)	58.00 (50.00, 63.00)	57.00 (50.00, 63.00)	59.00 (52.00, 64.00)	56.00 (49.00, 61.00)	56.00 (49.00, 62.00)	56.00 (50.00, 61.00)	56.00 (50.00, 61.00)
**Educational attainment**					**Educational attainment**			
*n* (%) missing	3105 (0.93)	1271 (0.70)	421 (0.51)	1413 (2.03)	226 (0.66)	46 (0.24)	13 (0.14)	167 (3.10)
Has a degree, *n* (%)	106 384 (31.83)	60 045 (33.07)	30 874 (37.23)	15 465 (22.18)	15 524 (45.58)	8991 (46.12)	4579 (49.91)	1954 (36.27)
**Household income**					**Household income**			
*n* (%) missing	46 127 (13.80)	22 274 (12.27)	8930 (10.77)	14 923 (21.40)	2979 (8.75)	1566 (8.03)	628 (6.84)	785 (14.57)
*n* (%) in each income category								
<£18k	62 329 (18.65)	32 501 (17.90)	13 772 (16.61)	16 056 (23.03)	3530 (10.36)	2072 (10.63)	827 (9.01)	631 (11.71)
£18k to £30 999	73 830 (22.09)	41 779 (23.01)	17 902 (21.59)	14 150 (20.29)	6.992 (20.53)	4083 (20.94)	1853 (20.20)	1056 (19.60)
£31k to £51 999	76 417 (22.86)	42 474 (23.39)	20 447 (24.66)	13 496 (19.36)	9401 (27.60)	5377 (27.58)	2557 (27.87)	1467 (27.23)
£52k to £100k	59 938 (17.93)	33 266 (18.32)	17 436 (21.03)	9236 (13.25)	9787 (25.80)	4893 (25.56)	2615 (28.50)	1189 (22.07)
>£100k	15 586 (4.66)	9294 (5.12)	4430 (5.34)	1862 (2.67)	2369 (6.96)	1414 (7.25)	695 (7.57)	260 (4.83)
**Living alone**					**Living alone**			
*n* (%) missing	981 (0.29)	464 (0.26)	190 (0.23)	327 (0.47)	51 (0.15)	24 (0.12)	11 (0.12)	16 (0.30)
*n* (%) answered yes	60 558 (18.12)	31 583 (17.39)	15 016 (18.11)	13 959 (20.02)	5162 (15.16)	2904 (14.90)	1410 (15.37)	848 (15.74)
**Married**					**Married**			
*n* (%) missing	954 (0.29)	464 (0.26)	191 (0.23)	299 (0.43)	56 (0.16)	27 (0.14)	12 (0.13)	17 (0.32)
*n* (%) answered yes	247 610 (74.08)	136 554 (75.20)	61 596 (74.29)	49 460 (74.08)	26 614 (78.14)	15 281 (78.38)	7199 (78.46)	4134 (76.74)
**Pollution (inverse distance to major road)**					**Pollution (inverse distance to major road)**			
*n* (%) missing	4540 (1.36)	2402 (1.32)	1188 (1.43)	950 (1.36)	396 (1.16)	216 (1.11)	108 (1.18)	72 (1.34)
*n* (%) in each quintile (Q1 = farthest from road)								
Qu1	70 159 (20.99)	38 825 (21.38)	17 428 (21.02)	13 906 (19.94)	6985 (20.51)	4057 (20.81)	1864 (20.32)	1064 (19.75)
Qu2	66 726 (19.96)	36 220 (19.95)	16 570 (19.98)	13 936 (19.99)	6810 (20.00)	3920 (20.11)	1834 (19.99)	1056 (19.60)
Qu3	64 998 (19.45)	35 267 (19.42)	15 923 (19.20)	13 808 (19.80)	6423 (18.86)	3638 (18.66)	1730 (18.86)	1055 (19.58)
Qu4	64 364 (19.26)	34 790 (19.16)	15 942 (19.23)	13 632 (19.55)	6740 (19.79)	3899 (20.00)	1836 (20.01)	1005 (18.65)
Qu5	63 440 (18.98)	34 083 (18.77)	15 866 (19.13)	13 491 (19.35)	6704 (19.68)	3765 (19.31)	1803 (19.65)	1136 (21.08)
**Social network (frequency of friend/family visits)**					**Social network (frequency of friend/family visits)**			
*n* (%) missing	1926 (0.58)	540 (0.30)	219 (0.26)	1167 (1.67)	208 (0.61)	28 (0.14)	7 (0.08)	173 (3.21)
*n* (%) in each category								
Almost daily	39 054 (11.68)	21 950 (11.89)	8534 (10.29)	8930 (12.81)	3244 (9.52)	1942 (9.96)	766 (8.35)	536 (9.95)
2 to 4 times/week	104 315 (31.21)	58 435 (32.18)	25 277 (30.48)	20 603 (29.55)	10 162 (29.84)	6081 (31.19)	2650 (28.88)	1431 (26.56)
About once a week	118 650 (35.50)	64 863 (35.72)	30 449 (36.72)	23 338 (33.47)	12 804 (37.59)	7270 (37.29)	3609 (39.34)	1925 (35.73)
About once a month	43 841 (13.12)	23 108 (12.73)	11 937 (14.40)	8796 (12.62)	1486 (5060)	2794 (14.33)	1455 (15.86)	811 (15.05)
Once every few months	21 252 (6.36)	10 717 (5.90)	5399 (6.51)	5136 (7.37)	2233 (6.56)	1219 (6.25)	607 (6.62)	407 (7.55)
Never or almost never	4581 (1.37)	2093 (1.15)	973 (1.17)	1515 (2.17)	322 (0.95)	154 (0.79)	73 (0.80)	85 (1.76)
No friends/family outside household	608 (0.18)	241 (0.13)	129 (0.16)	238 (0.34)	25 (0.07)	7 (0.04)	8 (0.09)	10 (0.19)
**Townsend deprivation score quintiles (Q1 = least deprived)**					**Townsend deprivation score quintiles (Q1 = least deprived)**			
*n* (%) missing	404 (0.12)	213 (0.12)	110 (0.13)	81 (0.12)	30 (0.09)	18 (0.09)	11 (0.12)	1 (0.02)
*n* (%) in each quintile								
Qu1	72 601 (21.72)	40 813 (22.48)	18 626 (22.46)	13 162 (18.88)	8 505 (24.97)	4862 (24.94)	2354 (25.66)	1289 (23.92)
Qu2	70 937 (21.22)	39 614 (21.82)	17 778 (21.44)	13 545 (19.43)	8092 (23.76)	4636 (23.78)	2217 (24.16)	1239 (23.00)
Qu3	68 974 (20.64)	37 969 (20.91)	17 106 (20.63)	13 899 (19.93)	7058 (20.72)	4128 (21.17)	1836 (20.01)	1094 (20.30)
Qu4	64 640 (19.34)	34 748 (19.14)	16 005 (19.30)	13 887 (19.92)	6125 (17.98)	3446 (17.68)	1679 (18.30)	1000 (18.56)
Qu5	56 671 (16.96)	28 230 (15.55)	13 292 (16.03)	15 149 (21.73)	4248 (12.47)	2405 (12.34)	1078 (11.75)	765 (14.20)
**Health behaviours**					**Health behaviours**			
**Alcohol binge (frequency of consuming ≥6 units)**					**Alcohol binge (frequency of consuming ≥6 units)**			
*n* (%) missing	233 298 (69.80)	124 677 (68.66)	55 235 (66.61)	53 386 (68.66)	12 053 (35.39)	6847 (35.12)	3183 (34.69)	2023 (37.55)
*n* (%) in each category								
Never	50 329 (49.87)	27 890 (49.01)	13 688 (49.45)	8751 (53.57)	10 214 (29.99)	5800 (29.75)	2741 (29.87)	1673 (31.05)
Less than monthly	24 772 (24.54)	14 030 (24.65)	6978 (25.21)	3764 (23.04)	5760 (16.91)	3273 (16.79)	1640 (17.87)	847 (15.72)
Monthly	8998 (8.92)	5287 (9.29)	2492 (9.00)	1219 (7.46)	2122 (6.23)	1275 (6.54)	566 (6.17)	281 (5.22)
Weekly	13 304 (13.18)	7835 (13.77)	3513 (12.69)	1956 (11.97)	3146 (9.24)	1895 (9.72)	818 (8.92)	433 (8.04)
Daily or almost daily	3526 (3.49)	1868 (3.28)	1011 (3.65)	647 (3.96)	763 (2.24)	405 (2.08)	227 (2.47)	131 (2.43)
**Alcohol frequency**					**Alcohol frequency**			
*n* (%) missing	290 (0.09)	99 (0.05)	39 (0.05)	152 (0.22)	6 (0.02)	3 (0.02)	1 (0.01)	2 (0.04)
*n* (%) in each category								
Daily/almost daily	71 942 (21.52)	40 332 (22.21)	18 523 (22.34)	13 087 (18.77)	8092 (23.76)	4600 (23.60)	2289 (24.95)	1203 (22.33)
3/4 times per week	80 933 (24.23)	46 916 (25.84)	20 272 (24.45)	13 805 (19.80)	9816 (28.82)	5941 (30.47)	2563 (27.93)	1212 (24.35)
1/2 times per week	87 653 (26.23)	48 286 (26.59	21 469 (25.89)	17 898 (25.67)	8660 (25.43)	5000 (25.65)	2314 (25.22)	1346 (24.98)
1/3 times per month	36 751 (11.00)	18 870 (10.39)	9405 (25.89)	8476 (12.16)	3520 (10.34)	1885 (9.67)	967 (10.54)	668 (12.40
Special occasions	34 947 (10.46)	16 716 (9.21)	8401 (10.13)	9830 (14.10)	2552 (7.49)	1300 (6.67)	686 (7.48)	566 (10.50)
Former	11 378 (3.40)	5460 (3.01)	2491 (3.00)	3427 (4.92)	714 (2.10)	296 (2.03)	175 (1.91)	143 (2.65)
Never	10 273 (3.07)	3048 (4.37)	2317 (2.79)	3048 (4.37)	698 (2.05)	370 (1.90)	180 (1.96)	148 (2.75)
**Energy intake (KJ on previous day)**					**Energy intake (KJ on previous day)**			
*n* (%) missing	189 083 (56.57)	100 172 (55.16)	44 135 (53.23)	44 776 (64.22)	11 859 (34.58)	19 495 (33.77)	3166 (34.51)	2109 (39.14)
Mean (SD)	8898.03 (3022.75)	9010.12 (3090.70)	8825.39 (2893.99)	8645(2975.42)	8988.99 (2941.18)	9092.83 (304.55)	8916.84 (2786.60)	8712.32 (2820.03)
**Salt intake (added to food)**					**Salt intake (added to food)**			
*n* (%) missing	37 (0.01)	9 (0.00)	10 (0.01)	18 (0.03)	1 (0.01)	1 (0.01)	0 (0)	0 (0)
*n* (%) in each category								
Never/rarely	189 549 (56.71)	104 889 (57.76)	47 369 (57.13)	37 291 (53.48)	20 621 (60.55)	11 949 (61.29)	5567 (60.68)	3105 (57.63)
Sometimes	92 412 (27.65)	49 925 (27.49)	22 985 (27.72)	19 502 (27.97)	8940 (26.25)	5065 (25.98)	2403 (26.19)	1472 (27.32)
Usually	37 826 (11.32)	19 820 (10.91)	9335 (11.26)	8671 (12.44)	3516 (10.32)	1963 (10.07)	938 (10.22)	615 (11.41)
Always	14 403 (4.31)	6944 (3.82)	3218 (3.88)	4241 (6.08)	980 (2.88)	518 (2.66)	266 (2.90)	196 (3.64)
**Smoking status**					**Smoking status**			
*n* (%) missing	1138 (0.34)	470 (0.26)	208 (0.25)	460 (0.66)	65 (0.19)	31 (0.16)	13 (0.14)	21 (0.39)
Ever smoker, *n* (%) answered yes	38 344 (44.39)	81 163 (44.70)	36 504 (44.02)	33 292 (47.75)	13 376 (39.27)	7787 (39.94)	3457 (37,68)	2132 (39.57)
**Cardiovascular risk and biomarkers**					**Cardiovascular risk and biomarkers**			
**Adiposity (BMI)**								
*n* (%) missing	1080 (0.32)	391 (0.22)	219 (0.26)	470 (0.67)	38 (0.11)	26 (0.13)	6 (0.07)	6 (0.11)
Mean (SD)	27.39 (4.75)	26.85 (4.33)	27.65 (4.86)	28.49 (5.42)	26.63 (4.26)	26.22 (3.94)	26.90 (4.42)	27.62 (4.84)
**Adiposity (waist circumference, cm)**					**Adiposity (waist circumference, cm)**			
*n* (%) missing	563 (0.17)	214 (0.12)	118 (0.14)	231 (0.33)	13 (0.04)	8 (0.04)	4 (0.04)	1 (0.02)
Mean (SD)	90.32 (14.50)	88.86 (12.78)	91.33 (13.61)	92.95 (14.51)	88.30 (12.67)	87.19 (12.17)	90.38 (12.88)	90.51 (13.60)
**Arterial stiffness (stiffness index, higher = stiffer)**					**Arterial stiffness (stiffness index, higher = stiffer)**			
*n* (%) missing	225 115 (67.35)	120 413 (66.31)	56 120 (67.68)	48 582 (69.68)	22 756 (66.82)	12 872 (66.03)	6164 (67.18)	3720 (69.04)
Median (Q1, Q3)	9.06 (6.91, 11.23)	8.95 (6.82, 11.17)	9.11 (7.00, 11.25)	9.30 (7.10, 11.38)	8.90 (6.78, 11.11)	8.78 (6.72, 11.01)	8.99 (6.80, 11.24)	9.13 (6.99, 11.20)
**Calcium (mmol/L)**					**Calcium (mmol/L)**			
*n* (%) missing	42 538 (12.73)	23 017 (12.68)	10 513 (12.68)	9008 (12.92)	4496 (13.20)	2551 (13.09)	1141 (12.44)	804 (14.92)
Mean (SD)	2.38 (0.09)	2.38 (0.09)	2.38 (0.09)	2.38 (0.10)	2.38 (0.10)	2.38 (0.09)	2.37 (0.09)	2.38 (0.09)
**Total cholesterol (mmol/L)**					**Total cholesterol (mmol/L)**			
*n* (%) missing	15 577 (4.66)	8116 (4.47)	3919 (4.73)	3542 (5.08)	1666 (4.89)	877 (4.50)	5388 (6.83)	368 (6.83)
Mean (SD)	5.71 (1.14)	5.72 (1.12)	5.71 (1.15)	5.69 (1.19)	5.72 (1.09)	5.72 (1.08)	5.72 (1.10)	5.74 (1.09)
**Diastolic BP (mmHg)**					**Diastolic BP (mmHg)**			
*n* (%) missing	22 054 (6.60)	11 684 (6.43)	5.386 (6.50)	4984 (7.15)	2227 (6.54)	1231 (6.31)	585 (6.38)	411 (7.63)
Mean (SD)	82.24 (10.66)	82.00 (10.58)	82.42 (10.70)	82.75 (10.78)	81.52 (10.42)	81.20 (10.30)	81.80 (10.45)	82.17 (10.70)
**Inflammation, CRP (mg/L)**								
*n* (%) missing	16 266 (4.87)	8449 (4.65)	4120 (4.97)	3697 (5.30)	1744 (5.12)	912 (4.68)	447 (4.87)	385 (7.15)
Median (Q1, Q3)	1.32 (0.66, 2.73)	1.18 (0.60, 2.40)	1.38 (0.68, 2.87)	1.70 (0.82, 3.55)	1.08 (0.55, 2.17)	0.99 (0.52, 1.96)	1.16 (0.60, 2.35)	1.32 (0.66, 2.69)
**Systolic BP (mmHg)**								
*n* (%) missing	22 059 (6.60)	11 687 (6.44)	5387 (6.50)	4985 (7.15)	2227 (6.54)	1231 (6.31)	585 (6.38)	411 (7.63)
Mean (SD)	140.15 (19.65)	140.13 (19.64)	139.37 (19.52)	140.90 (19.80)	137.52 (18.85)	137 (18.85)	136.88 (18.65)	137.52 (18.85)
**Medical diagnoses** ^c^					**Medical diagnosesc**			
**Atrial fibrillation**					**Atrial fibrillation**			
*n* (%) missing	0 (0)	0 (0)	0 (0)	0 (0)	0 (0)	0 (0)	0 (0)	0 (0)
*n* (%) with diagnosis	5822 (1.74)	2919 (1.61)	1479 (1.78)	1424 (2.04)	433 (1.27)	246 (1.26)	114 (1.24)	73 (1.35)
**Cardiovascular disease**					**Cardiovascular disease**			
*n* (%) missing	0 (0)	0 (0)	0 (0)	0 (0)	0 (0)	0 (0)	0 (0)	0 (0)
*n* (%) with diagnosis	30 856 (9.15)	15 173 (8.36)	7399 (8.92)	8014 (11.49)	2197 (6.45)	1218 (6.25)	611 (6.66)	367 (6.81)
**Cerebrovascular disease**					**Cerebrovascular disease**			
*n* (%) missing	0 (0)	0 (0)	0 (0)	0 (0)	0 (0)	0 (0)	0 (0)	0 (0)
*n* (%) with diagnosis	5280 (1.58)	2635 (1.30)	1194 (1.44)	1721 (2.47)	307 (0.90)	154 (0.79)	92 (1.00)	61 (1.13)
**Chronic kidney disease**					**Chronic kidney disease**			
*n* (%) missing	0 (0)	0 (0)	0 (0)	0 (0)	0 (0)	0 (0)	0 (0)	0 (0)
*n* (%) with diagnosis	3882 (1.16)	1783 (0.98)	952 (1.15)	1147 (1.16)	261 (0.77)	142 (0.73)	66 (0.72)	53 (0.98)
**Chronic lung disease**					**Chronic lung disease**			
*n* (%) missing	0 (0)	0 (0)	0 (0)	0 (0)	0 (0)	0 (0)	0 (0)	0 (0)
*n* (%) with diagnosis	3548 (1.06)	1328 (0.73)	825 (0.99)	1395 (2.00)	160 (0.47)	75 (0.38)	39 (0.43)	46 (0.85)
**Dementia**					**Dementia**			
*n* (%) missing	0 (0)	0 (0)	0 (0)	0 (0)	0 (0)	0 (0)	0 (0)	0 (0)
*n* (%) with diagnosis		20 (0.01)	13 (0.02)	12 (0.02)	0 (0)	0 (0)	0 (0)	0 (0)
**Diabetes**					**Diabetes**			
*n* (%) missing	0 (0)	0 (0)	0 (0)	0 (0)	0 (0)	0 (0)	0 (0)	0 (0)
*n* (%) with diagnosis	15 890 (4.75)	6934 (3.82)	4090 (4.93)	4866 (6.98)	992 (2.91)	447 (2.29)	302 (3.29)	243 (4.51)
**Head injury**					**Head injury**			
*n* (%) missing	0 (0)	0 (0)	0 (0)	0 (0)	0 (0)	0 (0)	0 (0)	0 (0)
*n* (%) with diagnosis	1486 (0.44)	824 (0.45)	325 (0.39)	337 (0.44)	95 (0.28)	54 (0.28)	27 (0.29)	14 (0.26)
**Mood disorder**					**Mood disorder**			
n (%) missing	0 (0)	0 (0)	0 (0)	0 (0)	0 (0)	0 (0)	0 (0)	0 (0)
*n* (%) with diagnosis	28 940 (8.66)	13 777 (7.59)	7412 (8.94)	7751 (11.12)	3376 (9.91)	1726 (8.85)	961 (10.47)	689 (12.79)
**Musculoskeletal condition**					**Musculoskeletal condition**			
*n* (%) missing	0 (0)	0 (0)	0 (0)	0 (0)	0 (0)	0 (0)	0 (0)	0 (0)
*n* (%) with diagnosis	144 216 (43.15)	76 561 (42.16)	34 175 (41.22)	33 480 (48.02)	14 132 (41.49)	8136 (41.73)	3713 (40.47)	2283 (42.37)
**Neurological condition**					**Neurological condition**			
*n* (%) missing	0 (0)	0 (0)	0 (0)	0 (0)	0 (0)	0 (0)	0 (0)	0 (0)
*n* (%) with diagnosis	48 794 (14.60)	24 045 (13.24)	12 097 (14.59)	12 652 (18.15)	5138 (15.09)	2744 (14.08)	1455 (15.86)	939 (17.43)
**Psychotic conditions**					**Psychotic conditions**			
*n* (%) missing	0 (0)	0 (0)	0 (0)	0 (0)	0 (0)	0 (0)	0 (0)	0 (0)
*n (*%) with diagnosis	779 (0.23)	299 (0.16)	208 (0.25)	272 (0.39)	31 (0.09)	21 (0.11)	7 (0.08)	3 (0.06)
**Mental health**					**Mental health**			
**Current depression score**					**Current depression score**			
*n* (%) missing	31 179 (9.33)	13 661 (7.52)	6569 (7.52)	10 949 (15.70)	2100 (6.17)	1078 (5.53)	493 (5.37)	529 (9.82)
Median (Q1, Q3)	1.00 (0.00, 2.00)	1.00 (0.00, 2.00)	1.00 (0.00, 2.00)	1.00 (0.00, 3.00)	1.00 (0.00, 2.00)	1.00 (0.00, 2.00)	1.00 (0.00, 2.00)	1.00 (0.00, 3.00)
**Neuroticism score**					**Neuroticism score**			
*n* (%) missing	62 404 (18.67)	29 652 (16.33)	14 363 (17.32)	18 389 (26.37)	5254 (15.43)	2776 (14.24)	1369 (14.92)	1109 (20.58)
Median (Q1, Q3)	4.00 (1.00, 6.00)	3.00 (1.00, 6.00)	4.00 (1.00, 6.00)	4.00 (2.00, 7.00)	3.00 (1.00, 6.00)	3.00 (1.00, 6.00)	3.00 (1.00, 6.00)	3.00 (1.00, 7.00)
**Traumatic events**					**Traumatic events**			
*n* (%) missing	225 491 (67.47)	120 692 (66.47)	53 243 (64.21)	51 556 (74.94)	10 687 (31.38)	6146 (31.53)	2808 (30.60)	1733 (32.16)
*n* (%) with ≥1 traumatic events	49 491 (45.51)	27 690 (45.47)	13 286 (44.77)	8515 (46.8)	10 564 (31.02)	6081 (31.19)	2809 (30.62)	1.674 (31.07)
**Worrier status**					**Worrier status**			
*n* (%) missing	8382 (2.51)	3964 (2.18)	1915 (2.31)	2503 (3.59)	730 (2.14)	386 (1.98)	194 (2.11)	150 (2.78)
*n* (%) answered yes	184 878 (55.32)	97 082 (53.46)	46 738 (56.37)	41 058 (58.89)	17 496 (51.37)	9639 (49.44)	4848 (52.84)	3009 (55.85)
**Medication**					**Medication**			
**Antihypertensive medication**					**Antihypertensive medication**			
*n* (%) missing	2251 (0.67)	847 (0.47)	394 (0.48)	1010 (1.45)	272 (0.80)	76 (0.39)	33 (0.36)	163 (3.03)
Any meds, *n* (%)	69 374 (20.76)	34 004 (18.73)	17 318 (20.89)	18 052 (25.89)	4210 (12.36)	2258 (11.58)	1.170 (12.75)	782 (14.51)
**Psychotropic medication**								
*n* (%) missing	9813 (2.94)	5120 (2.82)	2408 (2.90)	2285 (3.28)	942 (2.77)	519 (2.66)	262 (2.86)	161 (2.99)
Any meds, *n* (%)	28 834 (8.63)	12 687 (6.99)	7158 (8.63)	8989 (12.89)	1957 (5.75)	932 (4.78)	528 (5.75)	497 (9.22)

BMI, body mass index; BP, blood pressure; CF, cognitive function;CRP, C-reactive protein; MET, metabolic equivalent of task; mvPA, moderate-vigorous PA; PA, physical activity; Q, quartile; Qu, quintile; SD, standard deviation; meds, medication.

a Individuals were classified as active if they met ≥10 MET-hours of moderate to vigorous PA per week. However, they also reported levels of light PA (walking) which did not contribute to this classification. Total PA includes light PA as well as mvPA. Therefore, there is a subset of individuals who are non-missing on light PA, but missing on both moderate and vigorous PA. These individuals will have a value for total PA but be counted as missing PA classification.

b Global CF = mean of z scores on four tests (assuming at least two non-missing values).

c Medical diagnoses based on linked health records with positive diagnosis indicating diagnosis on or before baseline assessment date.

**Table 1b:** Cognitive outcomes for imaging subsample at follow-up

	Total sample	Active (≥10 MET hours mvPA/week)	Inactive (<10 MET hours of mvPA/week)	Missing PA status^a^
***n* (%) of sample**	34 058 (100.00)	19 495 (57.24)	9175 (26.94)	5338 (15.82)
**Original cognitive tests at follow-up**				
**Numerical memory (z score)**				
*n* (%) missing	10 573 (31.04)	6114 (31.36)	2718 (29.62	1741 (32.31)
Mean (SD)	−0.38 (0.95)	−0.39 (0.95)	−0.42 (0.95)	−0.38 (0.95)
**Pairs matching (z score)**				
*n* (%) missing	2587 (7.60)	1527 (7.83)	598 (6.52)	463 (8.57)
Mean (SD)	0.25 (1.06)	0.24 (1.06)	0.27 (1.05)	0.24 (1.05)
**Prospective memory**				
*n* (%) missing	2065 (6.06)	1225 (6.28)	473 (5.16)	367 (6.81)
n (%) correct on first attempt	26 839 (78.80)	15 810 (77.87)	7483 (81.56)	4176 (77.51)
**Reaction time (z score)**				
*n* (%) missing	2259 (6.63)	1339 (6.87)	518 (5.65)	402 (7.46)
Mean (SD)	0.04 (0.96)	0.05 (0.96)	0.03 (0.95)	−0.01 (0.97)
**Reasoning (z score)**				
*n* (%) missing	2638 (7.75)	1571 (8.06)	598 (6.52)	469 (8.70)
Mean (SD)	−0.20 (0.96)	−0.22 (0.95)	−0.10 (0.97)	−0.26 (0.98)
**Additional follow-up tests**				
**Matrix pattern completion (z score)**				
*n* (%) missing	11 118 (32.64)	6417 (32.92)	2868 (31.26)	1833 (34.02)
Mean (SD)	−0.19 (0.94)	−0.21 (0.92)	−0.12 (0.95)	−0.25 (0.94)
**Paired associate learning (z score)**				
*n* (%) missing	11 581 (34.00)	6702 (34.38)	2994 (32.63)	1885 (34.99)
Mean (SD)	−0.22 (0.83)	−0.23 (0.83)	− 0.17 (0.81)	−0.29 (0.85)
**Symbol digit substitution (z score)**				
*n* (%) missing	11 097 (32.58)	6410 (32.88)	2858 (31.15)	1829 (33.95)
Mean (SD)	−0.08 (0.96)	−0.10 (0.94)	−0.03 (0.94)	−0.12 (0.96)
**Tower rearranging (z score)**				
*n* (%) missing	11 311 (33.21)	6516 (33.42)	2927 (31.90)	1868 (34.67)
Mean (SD)	−0.12 (0.94)	−0.13 (0.93)	−0.09 (0.96)	−0.14 (0.96)
**Trails B-A, time (z score)**				
*n* (%) missing	11 730 (34.44)	6795 (34.86)	2996 (32.65)	1939 (35.99)
Mean (SD)	0.03 (0.96)	0.00 (0.96)	0.08 (0.94)	0.03 (0.95)
		**Trails A, time (z score)**		
*n* (%) missing	11 134 (32.69)	6435 (33.01)	2863 (31.20)	1836 (34.08)
Mean (SD)	0.04 (0.96)	0.02 (0.96)	0.08 (0.95)	0.05 (0.95)
		**Trails B, time (z score)**		
*n* (%) missing	11 730 (34.44)	6795 (34.86)	2996 (32.65)	1939 (35.99)
Mean (SD)	0.04 (0.96)	0.00 (0.96)	0.10 (0.95)	0.04 (0.95)
**Trails B, errors (z score)**				
*n* (%) missing	11 230 (32.97)	6493 (33.31)	2885 (31.44)	1852 (34.37)
Mean (SD)	0.99 (1.57)	0.96 (1.57)	1.05 (1.55)	0.96 (1.57)
**Composite CF measures**				
**Global CF (z score)** ^b^				
*n* (%) missing	2370 (6.96)	1407 (7.22)	539 (5.87)	424 (7.87)
Mean (SD)	−0.05 (0.56)	−0.06 (0.57)	−0.10 (0.56)	−0.05 (0.57)
**Processing speed composite (z score)** ^c^				
*n* (%) missing	11 168 (32.79)	6446 (33.06)	2879 (31.38)	1843 (34.21)
Mean (SD)	−0.03(0.72)	−0.06 (0.74)	−0.01 (0.73)	−0.06 (0.74)
**Executive function composite (z score)** ^d^				
*n* (%) missing	11 186 (32.84)	6461 (33.14)	2875 (31.34)	1850 (34.34)
Mean (SD)	−0.02 (0.72)	−0.04 (0.72)	0.03 (0.73)	−0.02 (0.72)
**Reasoning composite (z score)** ^e^				
*n* (%) missing	11 281 (33.12)	6158 (33.43)	2905 (31.66)	1858 (34.48)
Mean (SD)	−0.21 (0.79)	− 0.23 (0.77)	−0.12 (0.79)	−0.26 (0.81)
**Memory composite (z score)** ^f^				
*n* (%) missing	10 410 (30.57)	6024 (30.90)	2681 (29.22)	1705 (31.64)
Mean (SD)	−0.12 (0.62)	−0.13 (0.62)	−0.08 (0.61)	−0.12 (0.62)

CF, cognitive function; MET, metabolic equivalent of task; mvPA, moderate-vigorous PA; PA, physical activity; SD, standard deviation.

a Individuals were classified as active if they met ≥10 MET-hours of moderate to vigorous PA per week. However, they also reported levels of light PA (walking) which did not contribute to this classification. Total PA includes light PA as well as mvPA. Therefore, there is a subset of individuals who are non-missing on light PA, but missing on both moderate and vigorous PA. These individuals will have a value for total PA but be counted as missing PA classification.

b Global CF = mean of z scores on 10 tests assuming at least two non-missing values.

c Processing speed composite = mean of Digit Symbol Substitution and Reaction Time (assuming non-missing on both measures).

d Executive function composite = mean of Tower Rearranging, Trails A and Trails B completion time (assuming non-missing on two measures).

e Reasoning composite = mean of Reasoning test and Matrix Pattern Completion (assuming non-missing on both measures).

f Memory composite = mean of Pairs Matching, Numeric Memory and Paired Associate Learning (assuming non -missing on two measures).

The first set of regression models (Table 2) used CF data that was measured cross-sectionally with the PA measure. Cognitive scores at baseline were entered as the dependent variable and total self-reported PA in MET-hours per week as a continuous independent variable. Models were initially run without adjustment, and then adjusted according to the nearest approximation of the minimum sufficient adjustment set (listed in the Table 2 footnote).

**Table 2:** Cross-sectional regression models for baseline cognitive function

Exposure	Cognitive score	Unadjusted					Adjusted^a^				
		*n*	Estimate^b^	95% CI	*P* (uncorr)	*P* (FDR)^c^	*n*	Estimate^b^	95% CI	*P* (uncorr)	*P* (FDR)^c^
Total PA, self-report (MET hrs/week)	Reaction Time	305 294	0.000131	0.0000659, 0.0001962	.0001	.0001	29 810	−.000168	−.0004764, .0001403	.4471	.5365
	Pairs Matching	300 847	−0.0005355	−0.0006077, −0.004634	<.0001	<.0001	29 664	−.0001841	−.0004921, .0001239	.2855	.5365
	Reasoning	100 204	−0.0023377	−0.0024488, −0.0022266	<.0001	<.0001	12 438	−.0009303	−.001292, −.0005685	<.0001	<.0001
	Numeric Memory	31 854	−0.0012581	−0.014492, −0.0010669	<0.0001	<0.0001	3613	−0.0001427	−0.0008524, 0.0005669	0.6934	0.6934
	Global CF^d^	300 915	−0.0004846	−0.0005331, −0.0004362	<0.0001	<0.0001	29 695	−0.0001974	−0.000393, −0.00000017	0.0480	0.1440
	Prospective Memory	89 022	0.9981991	0.9979159, 0.998482	<0.0001	<0.0001	2548	0.9986968	0.9955481, 1.001856	0.4183	0.53652

CF, cognitive function; CI, confidence interval; FDR, false-discovery rate; MET, metabolic equivalent of task; PA, physical activity; uncorr, uncorrected., HDL, high-density lipoprotein; LDL, low-density lipoprotein.

a Adjusted for: alcohol binge, alcohol frequency, antihypertensive medication, apoe-e4 allele count, body mass index, cardiovascular disease diagnosis, dementia genetic risk score, diabetes diagnosis, distance to major road, friend and family visits, gender, HDL cholesterol, head injury diagnosis, household income, kidney disease diagnosis, kJ of energy, lLDL cholesterol, living alone status, manual work, mood disorder diagnosis, musculoskeletal diagnosis, neurological disorder diagnosis, neuroticism score, psychosis diagnosis, psychotropic medication, salt added to food, smoking status, Townsend deprivation score, trauma status, waist circumference, worrier status. Also adjusted for technical covariates used with genetic risk scores.

b All expressed as z score units (standardized mean difference), except Prospective Memory which is expressed as an odds ratio.

c Probability adjusted using the Simes–Benjamini–Hochberg method implemented in the Stata qqvalue package.

d Global CF = mean of z scores on four tests (assuming at least two non-missing values).

The second set of regression models (Table 3) used the CF variables pertaining to the imaging visit, making the analysis longitudinal by design. The included covariates were as above with the addition of follow-up duration, and both self-reported PA and the covariate values were again taken from baseline data. This set of models was also repeated using accelerometer-measured PA (which was acquired after baseline CF measurement and thus not used in cross-sectional models).

**Table 3:** Longitudinal regression models for follow-up cognitive function

Exposure	Cognitive score	Unadjusted					Adjusted^a^				
		*n*	Estimate^b^	95% CI	*P* (uncorr)	*P* (FDR)^c^	*n*	Estimate^b^	95% CI	*P* (uncorr)	*P* (FDR)^c^
Total PA, self−report (MET hours/week)	Reaction Time	30 153	−0.0000553	−0.0002922, 0.0001816	0.6474	0.6474	6816	0.0001664	−0.0004255, 0.0007584	0.5816	0.6394
	Pairs Matching	29 845	−0.000428	−0.0006911, −0.0001649	0.0014	0.0015	6780	−0.0001783	−0.000858, 0.0005014	0.6071	0.6394
	Reasoning	29 801	−0.0027912	−0.0030282, −0.0025542	<0.0001	<0.0001	6779	−0.0017126	−0.0022898,−0.001134	<0.0001	<0.0001
	Numerical Memory	22 321	−0.001635	−0.0019111, −0.0013588	<0.0001	<0.0001	5006	−0.0015375	−0.0022407,−0.0008344	0.6394	0.6394
	Symbol Digit Substitution	21 831	−0.0016325	−0.0019087, −0 .0013564	<0 .0001	<0 .0001	4886	−0 .0006917	−0 .0014112, 0 .0000277	0 .0595	0 .0893
	Paired Associate Learning	21 343	−0.0015357	−0.0017798, −0.0012917	<0.0001	<0.0001	4805	−0.0004867	−0.0011079, 0.0001346	0.1247	0.1727
	Tower Rearranging	21 626	−0.0011721	−0.0014512, −0.0008931	<0.0001	<0.0001	4865	−0.0004471	−0.0011614, 0.0002672	0.2198	0.2826
	Matrix Pattern Completion	21 804	−0.0019887	−0.0022625, −0.001715	<0.0001	<0.0001	4881	−0.0009316	−0.0016148, −0.0002483	0.0075	0.0193
	Prospective Memory	30 330	0.9971918	0.9965854 0.9977987	<0.0001	<0.0001	6840	0.9981081	0.9961615, 1.000058	0.0573	0.0893
	Trails A (time)	21 709	−0.0013174	−0.0015988,− 0.0010359	<0.0001	<0.0001	4874	−0.0007286	−0.0014642, 0.0000005	0.0518	0.0893
	Trails B (time)	21 225	−0.0019304	− 0.0022155,−0.0016453	<0.0001	<0.0001	4827	−0.0010314	−0.0017477, −0.0003152	0.0048	0.0173
	Trails B−A (time)	21 225	−0.0015662	−0.0018518,−0.0012806	<0.0001	<0.0001	4827	−0.0009099	−0.0016336, −0.0001862	0.0137	0.0274
	Trails B (errors)	21 698	−0.002139	−0.0026008, −0.0016773	<0.0001	<0.0001	4866	−0.0015567	.−0027763, −0.000337	0.0124	0.0274
	Processing Speed (comp)^d^	21 767	−0.0009248	−0.0011378, −0.0007117	<0.0001	<0.0001	4873	−0.0003291	−0.0008781, 0.0002199	0.2400	0.2880
	Executive Function (comp)^e^	21 742	−0.001489	−0.0017003, −0.0012777	<0.0001	<0.0001	4871	−0.0007267	−0.0012586 −0.0001947	0.0074	0.0193
	Reasoning (comp)^f^	21 655	−0.0024415	−0.0026714, −0.0022116	<0.0001	<0.0001	4866	−0.0013687	−0.0019174, −0.0008199	<0.0001	<0.0001
	Memory (comp)^g^	22 467	−0.0011893	−0.0013674, −0.0010112	<0.0001	<0.0001	5029	−0.0007394	−0.0011853, −0.0002936	0.0012	0.0054
	Global CF (comp)^h^	30 048	−0.0012926	−0.0014324, −0.0011529	<0.0001	<0.0001	6804	−0.0006555	−0.0010004, −0.0003107	0.0002	0.0012
Physical activity, accelerometery (milligravity units)	Reaction Time	14 307	0.0029557	0.0012409, 0.0046705	0.0007	0.0063	3935	0.002567	−0.0007845, 0.0059099	0.1334	0.8058
	Pairs Matching	14 164	−0.0009559	−0.0028609, 0.0009492	0.3254	0.3584	3919	0.0006546	−0.0046161, 0.0033069	0.7460	0.8952
	Reasoning	14 148	0.0009323	−0.0026586, 0.000794	0.2898	0.3478	3919	0.0001802	−0.0035365, 0.0031761	0.9162	0.9162
	Numerical Memory	9901	0.0019117	−0.0001713, 0.0039947	0.0720	0.1566	2837	0.000409	−0.003878, 0.0046959	0.8516	0.9162
	Symbol Digit Substitution	9683	0.0030884	0.0010321, 0.0051447	0.0032	0.0162	2772	−0.0018307	−0.0059394, 0.0022781	0.3824	0.8952
	Paired Associate Learning	9522	0.0015174	−0.0003052, 0.0033401	0.1027	0.1849	2731	−0.0006999	−0.0042253, 0.0028255	0.6971	0.8952
	Prospective Memory	14 392	1.006038	1.0007, 1.011405	0.0266	0.0798	3828	1.003531	0.9898692, 1.017381	0.6143	0.8952
	Tower Rearranging	9611	−0.0019001	−0.0039706, 0.0001704	0.0721	0.1566	2759	−0.0028300	−0.0070429, 0.0013830	0.1879	0.8058
	Matrix Pattern Completion	9670	−0.0009633	−0.0030134, 0.0010868	0.3570	0.3584	2768	−0.0009649	−0.0051264, 0.0031966	0.6494	0.8952
	Trails A (time)	9668	0.0030946	0.0010132, 0.005176	0.0036	0.0162	2766	−0.0010388	−0.0052679, 0.0031902	0.6301	0.8952
	Trails B (time)	9455	0.0026019	0.0004972, 0.0047067	0.0154	0.0554	2742	−0.0026095	−0.0067544, 0.0015354	0.2171	0.8058
	Trails B−A (time)	9455	0.0018798	−0.0002126, 0.0039721,	0.0783	0.1566	2742	−0.0023665	−0.006514, 0.0017809	0.2633	0.8058
	Trails B (errors)	9362	0.001587	−0.001800, 0.004974	0.3584	0.3584	2760	−0.0051376	−0.0117453, 0.0014702	0.1275	0.8058
	Processing Speed (comp)^d^	9653	0.0035774	0.0019994, 0.0051555	<0.0001	0.0002	2763	0.0005342	−0.0026049, 0.0036733	0.7386	0.8952
	Executive Function (comp)^e^	9650	0.0001975	−0.0029336, 0.0033287	0.1309	0.2142	2764	0.0001975	−0.0029336, 0.0033287	0.9016	0.9162
	Reasoning (comp)^f^	9612	−0.0010418	−0.0027634, 0.0006798	0.2356	0.3228	2763	0.0016841	−0.0051657, 0.0014390	0.2686	0.8058
	Memory (comp)^g^	9964	0.0009278	−0.0004027, 0.0022584	0.1717	0.2576	2848	−0.0006474	−0.0032057, 0.0019109	0.6198	0.8952
	Global CF (comp)^h^	14 284	0.0005975	−0.0004228, 0.0016178	0.2510	0.3227	3933	0.0007286	−0.0012778, 0.0027349	0.4765	0.8952

CF, cognitive function; CI, confidence interval; FDR, false-discovery rate; MET, metabolic equivalent of task; PA, physical activity; uncorr, uncorrected.; comp, composite.

a Adjusted for: alcohol binge, alcohol frequency, antihypertensive medication, apoe-e4 allele count, body mass index, cardiovascular disease diagnosis, dementia genetic risk score, diabetes diagnosis, distance to major road, friend and family visits, gender, HDL cholesterol, head injury diagnosis, household income, kidney disease diagnosis, kJ of energy, LDL cholesterol, living alone status, manual work, mood disorder diagnosis, musculoskeletal diagnosis, neurological disorder diagnosis, neuroticism score, psychosis diagnosis, psychotropic medication, salt added to food, smoking status, Townsend deprivation score, trauma status, waist circumference, worrier status. Also adjusted for technical covariates used with genetic risk scores.

b All expressed as z score units (standardized mean difference), except Prospective Memory which is expressed as an odds ratio.

c Probability adjusted using the Simes–Benjamini–Hochberg method implemented in the Stata qqvalue package.

d Processing speed composite = mean of Digit Symbol Substitution and Reaction Time (assuming non-missing on both measures).

e Executive function composite = mean of Tower Rearranging, Trails A and Trails B completion time (assuming non-missing on two measures).

f Reasoning composite = mean of Reasoning test and Matrix Pattern Completion (assuming non-missing on both measures).

g Memory composite = mean of Pairs Matching, Numerical Memory and Paired Associate Learning (assuming non-missing on two measures).

h Global CF = mean of z scores on 10 tests (assuming at least two non-missing values).

Diagnostic checks were performed for all models to ensure the assumptions for regression were met. *P*-values were two-tailed and false discovery rate (FDR) correction was used within groups of models that tested the same hypotheses, to maintain the false-positive rate at 0.05. All analyses were conducted on a complete-case basis and missing values were not imputed.

## Results

### Sample characteristics

Table 1a shows descriptive statistics for the variables specified in the conceptual model at baseline for both the entire sample, and the subsample who returned for imaging. The subsample who returned for imaging was on average younger, more active, less deprived and generally healthier at baseline than the overall sample. It is also apparent that, within the baseline data, those who were missing PA status were less educated, more deprived and generally less healthy than the overall sample, suggesting that missingness on the moderate-vigorous PA measures (which determined the PA groups) was not random. High missingness on some cognitive tests reflects that some tests were introduced at different stages within the baseline recruitment window. Table 1b shows the cognitive outcomes for the imaging sample at follow-up. Generally, the descriptive statistics suggested very small reductions in CF, of similar magnitude across the PA groups. The mean duration between baseline and follow-up was 8.94 years (SD 1.76).

### Effect of physical activity on cognitive function at baseline

Table 2 shows the cross-sectional regression results estimating the effect of PA, expressed in MET-hours per week, on CF, expressed in z-score units (with the exception of Prospective Memory, which is an odds ratio reflecting the odds of a correct response). The unadjusted models indicated a trivially small effect of PA on CF. For each measure of CF, the direction of the effect was negative (harmful), except for Reaction Time which was positive (protective). When models were adjusted for covariates, the effect sizes were similarly tiny and the confidence intervals were wider.

### Effect of physical activity on cognitive function at follow-up

Table 3 displays the longitudinal regression results estimating the effect of self-reported PA, expressed in MET-hours per week, on CF expressed in z-score units (with the exception of Prospective Memory, which is an odds ratio reflecting the odds of a correct response). Results for the same models repeated using accelerometry averages (expressed in milligravity units), as a continuous measure of device-measured PA, are displayed in the lower half of the same table.

Across the unadjusted self-reported PA models, the estimated effect of PA on CF was trivially small. The direction of the effect was negative (harmful) for all CF measures. After adjusting for covariates, effects remained trivially small in magnitude, and in the negative direction.

For device-measured PA, there were trivially small effects in unadjusted models for Reaction Time, Symbol Digit Substitution, Trails A time and Processing Speed composite, all in the positive (protective) direction. After adjusting for the specified covariates, effect sizes estimates remained trivially small, with wider confidence intervals.

### Sensitivity analysis

Because the adjusted models contained large numbers of covariates, results were potentially sensitive to bias arising from missing data. To examine this possibility, a sensitivity analysis was performed by repeating the unadjusted analyses, restricted to those participants who had full covariate data. The results are presented in Supplementary Tables S1 and S2 (available as Supplementary data at *IJE* online). There was very little difference in effect estimates, indicating that the unadjusted relationship between PA and CF was very similar among people with and without missing covariate data. Therefore, it is unlikely that observed results in adjusted models are being driven by missing data bias in the analytic sample.

## Discussion

In this study using a large cohort of middle-aged to early old-age adults of White British ancestry to estimate the causal effect of PA on CF, virtually no relationship was observed between these variables. Due to very large sample sizes, the effects were estimated with high precision (narrow confidence intervals); however, they were of trivially small magnitude, and became smaller after adjustment for covariates. This pattern of results was unexpected as it does not align with most of the recent literature that was synthesized and reviewed to construct the conceptual model informing this analysis.^11^ However, a minority of the synthesized studies also reported no association between PA and CF^25–28^ and, in common with two of these studies, the UK Biobank sample was younger at baseline than most of the other cohorts in which protective effects have been found. Taken together, our findings may suggest that observed associations in older baseline samples reflect reverse causation, whereby some participants had preclinical cognitive decline at baseline. In other words, physically slowing down may reflect a prodromal symptom of cognitive decline, rather than a cause of it. Indeed, recent evidence indicates that behavioural changes due to preclinical disease may account for observed exposure-outcome associations.^29–31^ Studies with younger samples at baseline, such as ours, reduce the risk that preclinical disease processes have begun. Another possible consequence of our sample’s relatively young age is that benefits of PA for CF have not yet been realized, and may yet be observable later in life when a greater degree of cognitive decline would be expected.^32^ Other potential explanations for our finding are considered below.

The UK Biobank sample is known not to be representative of the general population, with participants being more wealthy and educated and less likely to engage in unhealthy behaviours and experience negative health outcomes.^33^ When both exposure and outcome are related to participation in studies, this can lead to collider bias,^34^ and it is plausible that both higher levels of PA and better cognitive health influence participation and retention within UK Biobank. Indeed, the sample who returned for follow-up were more active, less deprived and healthier than the total sample. The implication is that the true relationship between PA and CF may be underestimated within UK Biobank due to participants’ better health, as has been demonstrated for other outcomes in recent studies.^35–36^ Furthermore, a recent analysis using UK Biobank indicated that analyses using socio-behavioural variables such as PA are particularly susceptible to participation bias.^37^ Taken together, our findings underline the importance of subjecting studies using UK Biobank to rigorous methodological scrutiny.

Finally, it is worth considering our results in the context of global public health guidance, which emphasizes that PA is just one of a broader suite of modifiable risk-factors against cognitive decline.^38^ Our results would support the notion that focusing on PA alone is unlikely to substantially reduce risk of decline.

## Strengths and limitations

The use of a DAG to inform the examination of the PA-CF relationship represents a novel contribution to the literature and, by following a protocol, was done to a standard of rigour and transparency that is not common within existing literature.^24^ However, the complexities of the rigorous model (dozens of nodes and hundreds of paths) posed practical limitations, such as being unable to interrogate its structural implications comprehensively using the available software, meaning that the plausible alternative variations of the specified model were not explored. Nor were the implied independencies of the model tested against the measured data, which is another way of assessing model fit.^39^

The use of UK Biobank data represents a trade-off in terms of strengths and limitations. The range and detail of measures available within this resource allowed a close approximation of the complex conceptual model to be estimated statistically. However, the internal and external validity of the findings are limited due to the selection bias within the sample, and the genetic ancestry restriction means results cannot be generalized beyond populations of White British ancestry.

Finally, total PA was selected as the exposure variable, as this had the fewest missing data relative to other categories of PA. However it is possible that the inclusion of ‘light PA’ within this has diluted the specific influence of moderate to vigorous PA, which has been observed to be associated with CF in a recent study using UK Biobank.^40^

## Conclusions

Due to limitations of both internal and external validity as discussed above, our results should be interpreted with caution. However, in the context of existing literature, the finding of no meaningful association between PA and CF aligns with other studies that had younger baseline samples, and may lend weight to the reverse causation hypothesis. Alternatively, the virtually null findings may reflect the suppression of true effects due to collider bias induced by the factors influencing participation and retention within the UK Biobank sample. Future research using UK Biobank can explore whether the hypothesized protective effect of PA on CF does emerge as the cohort matures and, if so, whether this effect is mediated by the hypothesized pathways via brain health.

## Ethics approval

Ethical approval was granted by the NHS Research Ethics Committee (16/NW/0274).

## Supplementary Material

dyad009_Supplementary_Data

## Data Availability

UK Biobank is an open access resource. Access procedures are described at [https://www.ukbiobank.ac.uk/enable-your-research]. The statistical analysis code for this study is available online at [https://osf.io/tngqh/].
