# Supplementary material for: Estimating the effect of physical activity on cognitive function within the UK Biobank cohort
Source: Int J Epidemiol. 2023 Feb 7;52(5):1592–611. doi: 10.1093/ije/dyad009 (PMC10555922; doi:10.1093/ije/dyad009)
Supplement: dyad009_Supplementary_Data [file dyad009_supplementary_data.docx]

**Supplementary Materials for:**

**Estimating the effect of physical activity on cognitive function within the UK Biobank cohort (Campbell & Cullen)**

**Supplementary Methods**

**Description of cognitive measures**

*Reaction Time*

Participants were shown pairs of cards on a screen and asked to press a button as quickly as possible when the two cards were identical. Twelve pairs were shown in total.

*Pairs Matching*

Participants were shown 12 cards onscreen simultaneously and were asked to recall the position of six matching pairs.

*Reasoning*

Participants were given two minutes to answer 13 multiple-choice verbal and numerical reasoning questions. UK Biobank refers to this as a fluid intelligence test; however some questions require crystallised abilities, and thus the task has been described as a reasoning test here.

*Numeric Memory*

Participants were presented with a string of numbers onscreen, and asked to enter them on a keypad in reverse order from memory, following a brief delay. This test was intended to require participants to mentally reverse the numbers, making it similar to backward digit span tasks. However, the stimuli were actually presented simultaneously rather than sequentially meaning the participants were able to achieve the correct response by recalling digit strings without reversing them if they read the numbers from right to left. This represents a forward digit span task which is an easier task, reflecting the attention/working memory domain.

*Prospective Memory*

Participants were presented with onscreen text informing them that at the end of the cognitive tests they would see four coloured symbols and be asked to touch the blue square. However, the instructions went on to inform the participant that they are to touch the orange circle instead. This required the participant to recall and respond in accordance with the true objective of the test.

*Trail-making Test*

Part A required participants to click on 25 numbered circles in ascending order, reflecting processing speed. Part B involved a similar task but switching between letters and numbers, reflecting processing speed plus executive function. Scores for each part reflect time taken in seconds to correctly click all circles. Additional scores comprising Part B time minus Part A time (as a more sensitive measure of executive function) and the number of errors made on part B were also analysed.

*Digit Symbol Substitution Test*

A grid of eight symbols, each corresponding to a number, was displayed onscreen. Participants used a keypad to enter the number corresponding to each symbol as it was presented onscreen, and scores reflect the total number of boxes correctly filled within two minutes. This task primarily reflects processing speed.

*Tower Rearranging Test*

Participants were presented with an onscreen illustration of three pegs, upon which three coloured hoops had been placed. They were then asked how many moves would be required to rearrange the hoops into another specified configuration. This test reflects executive function.

*Paired Associate Learning*

Participants were shown twelve pairs of words for 30 seconds in total, and after an interval were presented with the first word for ten of these pairs and asked to select the matching word from lists of four alternatives. This test reflects verbal declarative memory.

*Matrices*

Participants were presented with a series of matrix pattern blocks with an element missing and asked to select the element that best completed the pattern from a range of specified choices. This test reflects non-verbal reasoning.

**Description of covariates**

*Genetic*

A polygenic risk score for dementia (LDpred score based on Kunkle et al 2019 genome-wide association study of Alzheimer’s disease: <https://doi.org/10.1038/s41588-019-0358-2>) was standardised as a z-score and treated as a continuous variable. A variable reflecting APOE genotype (number of e4 alleles) was treated as continuous. Technical covariates related to genetic variables were also included in adjusted models (batch, chip and first eight principal components).

*Sociodemographic and local environment*

Age was calculated centrally by UK Biobank in whole years. Acculturation was represented by the number of years lived in the UK. Gender, ethnic group and country of origin were self-reported via touchscreen interview. Townsend deprivation index scores were derived centrally by UK Biobank from postcode of residence, and categorised into quintiles with one representing the least, and five the most, deprived. Income was treated as an ordinal variable reflecting which category (low to high) participants belonged to. Education was dichotomised to reflect whether participants had a university/college degree or not. A variable representing exposure to pollution was derived using the inverse distance from a person’s home address to a major road and split into quintiles with one representing the farthest distance and five the nearest. Living alone was recorded by touchscreen interview and treated as dichotomous (yes/no).

*Health behaviours, medical risk factors and physical measurements*

Past and current smoking habits were self-reported via touchscreen interview and a binary variable (ever vs never smoker) was derived. Sodium intake and alcohol frequency were also self-reported and treated as ordinal variables. Frequency of binge drinking (defined as greater than 6 units in one sitting) was recorded later using the web questionnaire treated as an ordinal variable. Biochemistry markers were assayed centrally from blood samples. Cholesterol (hdl, ldl and total) and calcium were treated as continuous variables expressed in mmol/L, and inflammation (serum C-reactive protein level, expressed in mg/L) was treated as ordinal. Body mass index was calculated as kg/m^2^ and treated as a continuous variable, and waist circumference was measured in cm and treated as continuous. Systolic and diastolic blood pressure were measured in mmHg and treated as continuous variables.

*Medical comorbidities and medications*

Relevant medical diagnoses indicated by the conceptual model were ascertained from linked health records or self-report interview data. Dichotomous variables were derived whereby ‘yes’ represented the participant receiving a diagnosis on or before the date of baseline assessment. The relevant diagnostic categories were: neurological conditions, dementia, diabetes, chronic kidney disease, head injury, cerebrovascular disease, chronic lung disease, musculoskeletal conditions, mood disorder, psychotic illness and cardiovascular disease. Dichotomous variables for antihypertensive and psychotropic medications were derived, coded ‘yes’ if the participant self-reported being on any medication within these categories.

*Mental health*

Trait anxiety was represented by a question asked at baseline (“Are you a worrier?”) and treated as a dichotomous variable (yes/no). Neuroticism was measured using 12 self-report questions at baseline (e.g., “Are you an irritable person?) and a continuous variable was derived representing the number of items the participant answered yes to. Experience of trauma/adverse experiences was measured using the web-based questionnaire (e.g., “When I was growing up, someone sexually molested me”, and a dichotomous variable representing any incidence of trauma was derived. Mood was measured using four questions regarding depression symptoms in the past two weeks, which were summed and treated as a continuous variable.

*Other*

Follow-up duration was calculated by subtracting the date of baseline assessment from the date of follow-up assessment and expressed in years.

**Matching of variables from conceptual model to UK Biobank data**

| **Concept** | **Biobank match** | **Link** |
| --- | --- | --- |
| Acculturation | Year moved to UK | <https://biobank.ndph.ox.ac.uk/showcase/field.cgi?id=3659> |
| Adiposity | Body mass index  Waist circumference | <https://biobank.ctsu.ox.ac.uk/crystal/field.cgi?id=21001>   <https://biobank.ctsu.ox.ac.uk/crystal/field.cgi?id=48> |
| Adverse experiences | Traumatic events | <https://biobank.ndph.ox.ac.uk/showcase/label.cgi?id=145> |
| Age | Age in years | <https://biobank.ndph.ox.ac.uk/showcase/field.cgi?id=21003> |
| Alcohol | Frequency   Binge | <https://biobank.ctsu.ox.ac.uk/crystal/field.cgi?id=1558>  <https://biobank.ctsu.ox.ac.uk/crystal/field.cgi?id=20416> |
| Brain health | Neuroimaging | <https://biobank.ndph.ox.ac.uk/showcase/label.cgi?id=100> |
| All medical diagnoses | First occurrences (pre baseline) | <https://biobank.ndph.ox.ac.uk/showcase/label.cgi?id=1712> |
| Calcium | Blood biochemistry | <https://biobank.ctsu.ox.ac.uk/crystal/field.cgi?id=30680> |
| Cardiovascular risk | Atrial fibrillation  Arterial stiffness  Blood pressure | <https://biobank.ctsu.ox.ac.uk/crystal/field.cgi?id=131350>  <https://biobank.ctsu.ox.ac.uk/crystal/field.cgi?id=21021>  <https://biobank.ctsu.ox.ac.uk/crystal/field.cgi?id=4079>  <https://biobank.ctsu.ox.ac.uk/crystal/field.cgi?id=4080> |
| Childhood IQ | Vocab measure from imaging visit | Not available for analysis |
| Childhood physical activity | Not measured | - |
| Cholesterol | Blood biochemistry | <https://biobank.ctsu.ox.ac.uk/crystal/field.cgi?id=30690> |
| Cognitive function | Baseline and follow-up tests | <https://biobank.ndph.ox.ac.uk/showcase/label.cgi?id=100026> |
| Diet | Energy consumed in previous 24hr | <https://biobank.ndph.ox.ac.uk/ukb/field.cgi?id=26002> |
| Education | Degree or not | <https://biobank.ndph.ox.ac.uk/ukb/field.cgi?id=6138> |
| Ethnicity | Ethnic category | <https://biobank.ndph.ox.ac.uk/showcase/field.cgi?id=21000> |
| Family history dementia/PD | Parent or sibling with diagnosis | <https://biobank.ndph.ox.ac.uk/ukb/label.cgi?id=100034> |
| Genetic risk | Dementia risk | <https://biobank.ndph.ox.ac.uk/ukb/label.cgi?id=263> |
| Inflammation | C-reactive protein | <https://biobank.ndph.ox.ac.uk/showcase/field.cgi?id=30710> |
| Living alone | Number in household | <https://biobank.ctsu.ox.ac.uk/crystal/field.cgi?id=709> |
| Manual work | Job involves manual/heavy work | <https://biobank.ctsu.ox.ac.uk/crystal/field.cgi?id=816> |
| Marital status | How are people in house related to participant | <https://biobank.ctsu.ox.ac.uk/crystal/field.cgi?id=6141> |
| Maternal smoking | Maternal smoking around birth | <https://biobank.ndph.ox.ac.uk/showcase/field.cgi?id=1787> |
| Physical activity | Baseline self-report scores  Accelerometry | <https://biobank.ndph.ox.ac.uk/showcase/label.cgi?id=54>  <https://biobank.ndph.ox.ac.uk/showcase/label.cgi?id=1009> |
| Pollution | Inverse distance to major road | <https://biobank.ctsu.ox.ac.uk/crystal/field.cgi?id=24012> |
| Psychotropic and anti hypertensive medication | Medication touchscreen questions;  psychotropics from verbal interview; cardiometabolic from touchscreen multiple choice | <https://biobank.ndph.ox.ac.uk/showcase/field.cgi?id=20003> |
| Socioeconomic | Household Income  Townsend deprivation index | <https://biobank.ctsu.ox.ac.uk/crystal/field.cgi?id=738>  <https://biobank.ctsu.ox.ac.uk/crystal/field.cgi?id=189> |
| Sex | Sex | <https://biobank.ndph.ox.ac.uk/showcase/field.cgi?id=31> |
| Smoking status | Tobacco history | <https://biobank.ctsu.ox.ac.uk/crystal/field.cgi?id=24012> |
| Social network | Frequency of friend/family visits | <https://biobank.ctsu.ox.ac.uk/crystal/field.cgi?id=1031> |
| Sodium | Salt added to food | <https://biobank.ctsu.ox.ac.uk/crystal/field.cgi?id=1478> |
| Trait anxiety | Neuroticism score  Are you a worrier | <https://biobank.ctsu.ox.ac.uk/crystal/field.cgi?id=20127> |

**Supplementary Results**

**Table S1:** Sensitivity analyses for cross-sectional models

| **Exposure** | **Cognitive score** | **Unadjusted, within the total sample** | | | | | **Unadjusted, within the sample that had complete covariate data** | | | | |
| --- | --- | --- | --- | --- | --- | --- | --- | --- | --- | --- | --- |
| Total PA, self-report (MET hrs/week) |  | **n** | **Estimate^a^** | **95% CI** | **P (uncorr)** | **p (FDR)^b^** | **n** | **Estimate^a^** | **95% CI** | **p (uncorr)** | **p (FDR)^b^** |
|  | Reaction Time | 305,294 | .000131 | .0000659, .0001962 | .0001 | <.0001 | 29,810 | .0001306 | -.0001092, .0003704 | .2857 | .3429 |
|  | Pairs Matching | 300,847 | -.0005355 | -.0006077, -.004634 | <.0001 | <.0001 | 29,664 | -.0004247 | -.0006967, -.0001528 | .0022 | .0033 |
|  | Reasoning | 100,204 | -.0023377 | -.0024488, -.0022266 | <.0001 | <.0001 | 12,438 | -.002656 | -.002994, -.0023179 | <.0001 | <.0001 |
|  | Numeric Memory | 31,854 | -.0012581 | -.014492, -.0010669 | <.0001 | <.0001 | 3,613 | -.0013487 | -.001991, -.0007064 | <.0001 | <.0001 |
|  | Global CF^c^ | 300,915 | -.0004846 | -.0005331, -.0004362 | <.0001 | <.0001 | 29,695 | -.0005834 | -.0007574, -.0004093 | <.0001 | <.0001 |
|  | Prospective Memory | 89,022 | .9981991 | .9979159, .998482 | <.0001 | <.0001 | 2,548 | .9986968 | .9955481, 1.001856 | .4183 | .4183 |

CF, cognitive function; CI, confidence interval; FDR, false discovery rate; MET, metabolic equivalent of task; PA, physical activity; uncorr, uncorrected.
a. All expressed as z score units (standardised mean difference), except Prospective Memory which is expressed as an odds ratio.
b. Probability adjusted using the Simes-Benjamini-Hochberg method implemented in the Stata qqvalue package.
c. Global CF = mean of z scores on four tests (assuming at least two non-missing values).

**Table S2:** Sensitivity analyses for longitudinal models

| **Exposure** | **Cognitive score** | **Unadjusted, within the total sample** | | | | | **Unadjusted, within the sample that had complete covariate data** | | | | |
| --- | --- | --- | --- | --- | --- | --- | --- | --- | --- | --- | --- |
| Total PA, self-report (MET hrs/week) |  | ***n*** | **Estimate^a^** | **95% CI** | ***p* (uncorr)** | ***p* (FDR)**^b^ | ***n*** | **Estimate^a^** | **95% CI** | ***p* (uncorr)** | ***p* (FDR)**^b^ |
|  | Reaction time | 30,153 | -.0000553 | -.0002922, .0001816 | .6474 | .6474 | 6,816 | .0000752 | -.0006065, .0004562 | .7816 | .7816 |
|  | Pairs matching | 29,845 | -.000428 | -.0006911, -.0001649 | .0014 | .0015 | 6,780 | -.0003613 | -.0009561, .0002335 | .2338 | .2476 |
|  | Prospective Memory | 30,330 | .9971918 | .9965854, .9977987 | <.0001 | <.0001 | 6,840 | . 9971716 | .9955542, .9987915 | .0006 | .0007 |
|  | Reasoning | 29,801 | -.0027912 | -.0030282, -.0025542 | <.0001 | <.0001 | 6,779 | -.0032087 | -.0037301, -.0026873 | <.0001 | <.0001 |
|  | Numeric Memory | 22,321 | -.001635 | -.0019111, -.0013588 | <.0001 | <.0001 | 5,006 | -.0021687 | -.0028092, -.0015282 | <.0001 | <.0001 |
|  | Symbol Digit Substitution | 21,831 | -.0016325 | -.0019087, -.0013564 | <.0001 | <.0001 | 4,886 | -.0017565 | -.0023865, -.0011266 | <.0001 | <.0001 |
|  | Paired Associate Learning | 21,343 | -.0015357 | -.0017798, -.0012917 | <.0001 | <.0001 | 4,805 | -.0014098 | -.0019326, -.0008871 | <.0001 | <.0001 |
|  | Tower Rearranging | 21,626 | -.0011721 | -.0014512, -.0008931 | <.0001 | <.0001 | 4,865 | -.001141 | -.0017697, -.0005123 | .0004 | .0005 |
|  | Matrix pattern completion | 21,804 | -.0019887 | -.0022625, -.001715 | <.0001 | <.0001 | 4,881 | -.0021046 | -.0027152, -.0014941 | <.0001 | <.0001 |
|  | Trails A (time) | 21,709 | -.0013174 | -.0015988, -.0010359 | <.0001 | <.0001 | 4,874 | -.0015953 | -.0022335, -.0009571 | <.0001 | <.0001 |
|  | Trails B (time) | 21,225 | - .0019304 | -.0022155, -.0016453 | <.0001 | <.0001 | 4,827 | - .0023499 | -.0029889, -.0017108 | <.0001 | <.0001 |
|  | Trails B – A (time) | 21,225 | -.0015662 | -.0018518, -.0012806 | <.0001 | <.0001 | 4,827 | -.0019600 | -.002588, -.0013319 | <.0001 | <.0001 |
|  | Trails B (errors) | 21,698 | -.002139 | -.0026008, -.0016773 | <.0001 | <.0001 | 4,866 | -.0027761 | -.0037931, -.001759 | <.0001 | <.0001 |
|  | Processing Speed (comp)^c^ | 21,767 | -.0009248 | -.0011378, -.0007117 | <.0001 | <.0001 | 4,873 | -.000999 | -.0014791, -.000519 | <.0001 | <.0001 |
|  | Executive Function (comp)^d^ | 21,742 | -.001489 | -.0017003, -.0012777 | <.0001 | <.0001 | 4,871 | -.0016902 | -.002165, -.0012153 | <.0001 | <.0001 |
|  | Reasoning (comp)^e^ | 21,655 | -.0024415 | -.0026714, -.0022116 | <.0001 | <.0001 | 4,866 | -.002725 | -.0032274, -.0022226 | <.0001 | <.0001 |
|  | Memory (comp)^f^ | 22,467 | -.0011893 | -.0013674, -.0010112 | <.0001 | <.0001 | 5,029 | -.0012647 | -.0016617, -.0008677 | <.0001 | <.0001 |
|  | Global CF (comp)^g^ | 30,048 | -.0012926 | -.0014324, -.0011529 | <.0001 | <.0001 | 6,804 | -.0006555 | -.0010004, -.0003107 | .0002 | .0003 |
| **Exposure** | **Cognitive score** | **Unadjusted, within the total sample** | | | | | **Unadjusted, within the sample that had complete covariate data** | | | | |
| Physical Activity, accelerometry (milligravity units) |  | ***n*** | **Estimate^a^** | **95% CI** | ***p* (uncorr)** | ***p* (FDR)^b^** | ***n*** | **Estimate^a^** | **95% CI** | ***p* (uncorr)** | ***p* (FDR)^b^** |
|  | Reaction time | 14,307 | .0029557 | .0012409, .0046705 | .0007 | .0063 | 3,935 | .0027609 | -.0004749, .0059968 | .1334 | .8058 |
|  | Pairs matching | 14,164 | -.0009559 | -.0028609, .0009492 | .3254 | .3584 | 3,919 | -.0013041 | -.0049577, .0023496 | .7460 | .8393 |
|  | Prospective Memory | 14,392 | 1.006038 | 1.0007, 1.011405 | .0266 | .0798 | 3,828 | 1.001839 | .9904113, 1.013399 | .9162 | .9162 |
|  | Reasoning | 14,148 | .0009323 | -.0026586, .000794 | .2898 | .3478 | 3,919 | -.000163 | -.0033863, .0030603 | .8516 | .9017 |
|  | Numeric Memory | 9,901 | .0019117 | -.0001713, .0039947 | .0720 | .1566 | 2,837 | .0019519 | -.0020293, .0059331 | .3824 | .8393 |
|  | Symbol Digit Substitution | 9,683 | .0030884 | .0010321, .0051447 | .0032 | .0162 | 2,772 | .0004748 | -.0034015, .0043512 | .6971 | .8393 |
|  | Paired Associate Learning | 9,522 | .0015174 | -.0003052, .0033401 | .1027 | .1849 | 2,731 | .0013632 | -.0019934, .0047198 | .6143 | .8393 |
|  | Tower Rearranging | 9,611 | -.0019001 | -.0039706, .0001704 | .0721 | .1566 | 2,759 | -.0048043 | -.0087284, -.0008802 | .1879 | .8058 |
|  | Matrix pattern completion | 9,670 | -.0009633 | -.0030134, .0010868 | .3570 | .3584 | 2,768 | -.0012759 | -.0051684, .0026166 | .6494 | .8393 |
|  | Trails A (time) | 9,668 | .0030946 | .0010132, .005176 | .0036 | .0162 | 2,766 | .0008261 | -.0031038, .004756 | .6301 | .8393 |
|  | Trails B (time) | 9,455 | .0026019 | .0004972, .0047067 | .0154 | .0554 | 2,742 | .0023579 | -.0016326, .0063484 | .2171 | .8058 |
|  | Trails B – A (time) | 9,455 | .0018798 | -.0002126, .0039721 | .0783 | .1566 | 2,742 | .0022594 | -.0016632, .0061819 | .2633 | .8058 |
|  | Trails B (errors) | 9,362 | .001587 | -.001800, .004974 | .3584 | .3584 | 2,760 | .0037901 | -.0025624, .0101426 | .1275 | .8058 |
|  | Processing Speed (comp)^c^ | 9,653 | .0035774 | .0019994, .0051555 | <.0001 | .0002 | 2,763 | .0014516 | -.0014919, .0043952 | .7386 | .8393 |
|  | Executive Function (comp)^d^ | 9,650 | .0011996 | -.000357, .0027562 | .1309 | .2142 | 2,764 | -.0006113 | -.0035482, .0023255 | .6832 | .8396 |
|  | Reasoning (comp)^e^ | 9,612 | -.0010418 | -.0027634, .0006798 | .2356 | .3227 | 2,763 | -.0021697 | -.0053804, .0010411 | .2686 | .8058 |
|  | Memory (comp)^f^ | 9,964 | .0009278 | -.0004027, .0022584 | .1717 | .2576 | 2,848 | .0007733 | -.0016947, .0032413 | .6198 | .8393 |
|  | Global CF (comp)^g^ | 14,284 | .0005975 | -.0004228, .0016178 | .2510 | .3227 | 3,933 | .0007286 | -.0012778, .0027349 | .4765 | .8393 |

CF, cognitive function; CI, confidence interval; FDR, false discovery rate; MET, metabolic equivalent of task; PA, physical activity; uncorr, uncorrected.
a. All expressed as z score units (standardised mean difference), except Prospective Memory which is expressed as an odds ratio.
b. Probability adjusted using the Simes-Benjamini-Hochberg method implemented in the Stata qqvalue package.
c. Processing speed composite = mean of Digit Symbol Substitution and Reaction Time (assuming non-missing on both measures).
d. Executive function composite = mean of Tower Rearranging, Trails A and Trails B completion time (assuming non-missing on two measures).
e. Reasoning composite = mean of Reasoning test and Matrix Pattern Completion (assuming non-missing on both measures).
f. Memory composite = mean of Pairs Matching, Numeric Memory and Paired Associate Learning (assuming non -missing on two measures).
g. Global CF = mean of z scores on ten tests (assuming at least two non-missing values).
